# Detection of *Vibrio cholerae *and *Acanthamoeba *species from same natural water samples collected from different cholera endemic areas in Sudan

**DOI:** 10.1186/1756-0500-4-109

**Published:** 2011-04-07

**Authors:** Salah Shanan, Hadi Abd, Ingela Hedenström, Amir Saeed, Gunnar Sandström

**Affiliations:** 1 Karolinska Institute, Department of Laboratory Medicine, Division of Clinical Microbiology, Karolinska University Hospital, Huddinge, SE-141 86 Stockholm, Sweden; 2 Swedish Institute for Infectious Disease Control, SE-17182 Solna, Sweden; 3 University of Medical Sciences and Technology, Faculty of Medical Laboratory Sciences, Khartoum, Sudan

## Abstract

**Background:**

*Vibrio cholerae *O1 and *V. cholerae *O139 infect humans, causing the diarrheal and waterborne disease cholera, which is a worldwide health problem. *V. cholerae *and the free-living amoebae *Acanthamoeba *species are present in aquatic environments, including drinking water and it has shown that *Acanthamoebae *support bacterial growth and survival. Recently it has shown that *Acanthamoeba *species enhanced growth and survival of *V. cholerae *O1 and O139. Water samples from different cholera endemic areas in Sudan were collected with the aim to detect both *V. cholerae *and *Acanthamoeba *species from same natural water samples by polymerase chain reaction (PCR).

**Findings:**

For the first time both *V. cholerae *and *Acanthamoeba *species were detected in same natural water samples collected from different cholera endemic areas in Sudan. 89% of detected *V. cholerae *was found with *Acanthamoeba *in same water samples.

**Conclusions:**

The current findings disclose *Acanthamoedae *as a biological factor enhancing survival of *V. cholerae *in nature.

## Background

*Vibrio cholerae *species are widely distributed in aquatic environments [1]. They comprise nearly 200 serogroups based on the O antigenic structures [2]. *V. cholerae *O1 and *V. cholerae *O139 infect humans, causing the diarrheal and waterborne disease cholera [3], which is a worldwide health problem.

*V. cholerae *inhabits aquatic environments and human intestines [4], and cholera outbreaks are associated with contaminated food and water supplies. The seasonality of cholera has been associated with physical and biological factors [5]; however, many factors affect the survival of *V. cholerae *in aquatic environments such as attachment to plankton, loss to predators [6].

*Acanthamoebae *are free-living protozoa distributed worldwide in nature [7,8] and may affect the survival of *V. cholerae*. It is well known that *V. cholerae *and *Acanthamoeba *species are present in aquatic environments, including drinking water [9-11] and the use of water with poor microbiological quality increases the risk of human illness since acanthamoebae and bacteria are involved in complex interactions important to medical and environmental microbiology. It is known that *acanthamoebae *benefit from extracellular bacteria as food, which may enhance survival of the amoebae in different environments. In contrast, the role of *Acanthamoebae *as hosts for bacteria has been proposed for many pathogenic bacteria [12-24].

Recent publications [25-28] showed that *Acanthamoeba *species enhanced growth and survival of *V. cholerae *O1 and O139 in laboratory microcosm co-culture experiments, but no study has so far been published on the detection of both *V. cholerae *and *Acanthamoeba *from similar sites. In this context, cholera is endemic and poses a persistent threat to the public health in Sudan. It has been shown that cholera outbreaks, which occurred between April and August 2006 caused 6254 cases including 204 deaths with a case fatality rate of 3.2% in Northern Sudan [29].

In this paper, water samples from different cholera endemic areas in Sudan were collected with the aim to detect both *V. cholerae *and *Acanthamoeba *species from similar sites of collected natural water samples by polymerase chain reaction (PCR).

## Materials and methods

### Sample collection

Four hundred water samples collected from 4 states in Sudan previously known as foci of *V. cholerae*. The states are Gadarif, Juba, Kordofan and Khartoum. 128 samples were from zeers (home pots), 167 from hafirs (a hafir is an underground reservoir designed for storing rain water carried by streams and used for domestic water supply and for agricultural purposes in rural areas in the Sudan), and 66 from water tanks and 39 from lakes.

### DNA extraction

50 ml water was centrifuged for 10 min at 4000 rpm and the pellets were used for DNA extraction using Qiagen DNA mini kit (Qiagen, Valencia, CA, USA).

### DNA amplification

In the first reaction two primers sets were used. One set, referred to as the AcU primer 5'- GGC CCA GAT CGT TTA CCG TGA A-3' and the Ac L primer 5'-TCT CAC AAG CTG CTA GGG GAG TCA-3' and in the second reaction also two primers sets were used, one set referred to as the VCT-1 primer 5'-ACA GAG TGA GTA CTT TGA CC-3' and the VCT-2 primer 5' ATA CCA TCC ATA TAT TTG GGA G-3' PCR were carried out for the both reactions in a final volume of 20 μl containing each primer at a concentration of 0.3 μM, 1.0× PCR golden buffer, 200 μM deoxyribonucleoside triphospate, 1.2 mM Mgcl_2_, 1.25 U/50 μl of Ampli Taq Gold (Sigma).

### Gel analysis of PCR product

PCR condition were: 32 cycles of 95°C (denaturation) for 4 min, 55°C (annealing) for 20 sec, and 72°C for 10 sec (extension). PCR products were analyzed by electrophoresis on agarose gel in 1× TBE buffer (Tri base, boric acid and EDTA (pH 8.0). The gel was stained in 0.1% SYBR Green bath, visualized by UV translumination, and photographed using Polaroid films. DNA fragment 487 bp for *Acanthamoeba *was obtained in the first reaction and DNA fragment 308 bp for *Vibrio cholerae *toxin was obtained in the second reaction.

### Statistical analysis

χ2 test was performed for comparative statistical analysis of together-and alone-detected microorganisms to show the significant existence of alone *V. cholerae *or that found with *Acanthamoeba*.

## Results and discussion

*V. cholerae *O1 and *V. cholerae *O139 are widely distributed in aquatic environments [17] causing the diarrheal and waterborne disease cholera [22]. *V. cholerae *and *Acanthamoeba *species are present in aquatic environments, including drinking water [9,16,19]. A number of studies report that free-living amoebae (FLA) support survival of pathogenic bacteria [19] and more studies are still needed on distribution of *V. cholerae *and FLA in nature [30]. In the current study we collected water samples from endemic areas in Sudan to detect *V. cholerae *and *Acanthamoeba *species in same natural water samples by PCR targeting cholera toxin gene (*toxA*) and *Acanthamoeba *18 S RNA gene.

A total of 400 water samples were examined by PCR to detect *V. cholerae *toxin gene (*toxA*) and *Acanthamoeba *18 S RNA gene. The result showed that 8 water samples numbered 8, 117, 121, 150, 156, 160, 193, and 213 contained both *V. cholerae *and *Acanthamoeba *(table 1). Furthermore, it was found that only one water sample contained *V. cholerae *(number 54) compared to 13 samples numbered 24, 46, 70, 84, 87, 128, 177, 202, 259, 266, 287, 319, and 397, which contained *Acanthamoeba *only (table 1).

**Table 1:** PCR result showing positive *Acanthamoeba *and *V. cholerae *in same sample and *Acanthamoeba *or *V. cholerae *alone

Sample number	Region	Source	*V. cholerae*	*Acanthamoebae*
8	Gadarif	zeer	+ve	+ve

24	Gadarif	zeer	-ve	+ve

46	Gadarif	hafir	-ve	+ve

54	Gadarif	hafir	+ve	-ve

70	Gadarif	hafir	-ve	+ve

84	Gadarif	tank	-ve	+ve

87	Gadarif	tank	-ve	+ve

117	Gadarif	tank	+ve	+ve

121	Gadarif	tank	+ve	+ve

128	Gadarif	tank	-ve	+ve

150	Juba	lake	+ve	+ve

156	Juba	lake	+ve	+ve

160	Juba	lake	+ve	+ve

177	Juba	zeer	+ve	+ve

193	Juba	zeer	+ve	+ve

202	Juba	zeer	-ve	+ve

213	Khartoum	lake	+ve	+ve

259	Khartoum	zeer	-ve	+ve

266	Khartoum	zeer	-ve	+ve

287	Kordofan	hafir	-ve	+ve

319	Kordofan	hafir	-ve	+ve

397	Kordofan	hafir	-ve	+ve

Analyzing presence of detected microorganisms showed that the detected number of together- and alone-identified microorganisms (amoebae and bacteria) (table 2) was significantly differed (*p *value of χ2 was < 0.05). *V. cholerae *needs to be found with other microorganisms such as *Acanthamoebae *a finding disclosed by this study since 89% of detected *V. cholerae *was found with *Acanthamoebae *compared to 11% *V. cholerae*, which was found alone. As regards amoebae 38% of *Acanthamoebae *was found with *V. cholerae *and 62% was found alone. Moreover, prevalence of *V. cholerae *alone was 0.25% and that of *Acanthamoeba *alone was 3.25%, while prevalence of both *Acanthamoeba *and *V. cholerae *was 2% (table 2). Taken together, this clearly shows that *Acanthamoeba *and *V. cholerae *can be isolated at similar sites but it does not disclose interaction between them. However, in previous studies it has been found that *Acanthamoeba *and *V. cholerae *interact in beneficial ways for both microorganisms and it could thus be speculated that such interaction is important for the microorganisms also in nature. [12-24].

**Table 2:** Prevalence of detected microorganisms

Detected microorganisms	Positive	Prevalence %
*Acanthamoeba *and *V. cholerae*	8/400	2

*Acanthamoeba *only	13/400	3.25

*V. cholerae *only	1/400	0.25

For the first time this study show that both *V. cholerae *and *Acanthamoeba *species can be detected in the same natural water samples collected from different cholera endemic areas in Sudan. 89% of detected *V. cholerae *was found with *Acanthamoeba *and 11% was found alone. Taken together role of *Acanthamoeba *species in survival of *V. cholerae *[18,25,26,31] may strongly disclose *Acanthamoedae *as a biological factor enhancing survival of *V. cholerae *in nature.

*Acanthamoebae *support bacterial survival and growth [12,25,26,31] and save the bacteria from the effects of chlorination [5], antibodies [5] and antibiotics [12,25,26,31], increasing the risk of human illness caused by bacteria or *Acanthamoebae*. Accordingly, a need to find an effective means of detection and killing of both *Acanthamoeba *and bacteria is warranted to reduce the risk of spread of *V. cholerae*.

## Conclusions

89% of detected *V. cholerae *was found with *Acanthamoeba *disclosing *Acanthamoedae *as a biological factor enhancing survival of *V. cholerae *in nature.

## Competing interests

The authors declare that they have no competing interests.

## Authors' contributions

SS collected the samples extracted DNA and perform PCR and gel electrophoresis. HA conceived the study, draft the manuscript and performed the statistical analysis. IH guided PCR and gel electrophoresis. AS participated in writing. GS participated in writing and critical reading. All authors read and approved the final manuscript.
